# Green synthesis of ice-templated lotus starch–bentonite cryogels for sustainable and energy-efficient VOC capture

**DOI:** 10.1039/d5ra07007d

**Published:** 2025-11-26

**Authors:** Huarong Liu, Hong Liu, Ning An, Kui Yang, Zhongwei Liu

**Affiliations:** a Sichuan College of Architectural Technology China liuhong@scac.edu.cn

## Abstract

Sustainable adsorbents for industrial volatile organic compound (VOC) control are urgently needed to balance efficiency and operational economy. This report describes an eco-friendly strategy for fabricating hierarchical porous bentonite (Bt) *via* green ice-templating using lotus root starch as a biomass crosslinker. The optimized material exhibits a well-defined lamellar-bridging network with a specific surface area of 71.3 m^2^ g^−1^ and a pore volume of 0.118 cm^3^ g^−1^.This adsorbent exhibits high toluene capacity (53.8 mg g^−1^; 4.7× raw Bt) with polarity-driven selectivity for chlorobenzene (54.3 mg g^−1^) > toluene > benzene (46.2 mg g^−1^), facilitated by multiscale transport and cation-polar molecule interactions. Notably, the material enables humidity-resistant operation and low-energy regeneration at 110 °C while retaining 78% capacity after 10 cycles. With its natural composition, low-energy synthetic route avoiding calcination, and efficient regeneration profile, this work provides a sustainable and cost-effective material platform toward next-generation low-carbon air pollution control systems.

## Abstract 2

A lotus starch–bentonite porous monolith, fabricated *via* green ice-templating, has a multi-porous structure and demonstrates polarity-driven VOC selectivity, enabling efficient capture and low-energy regeneration.

## Graphical abstract

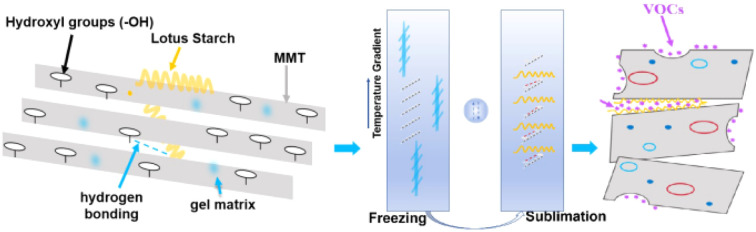

## 1 Introduction

Volatile organic compounds (VOCs), which comprise more than 300 species with boiling points below 250 °C at atmospheric pressure, represent critical air pollutants derived from industrial processes, such as petrochemical refining and solvent applications.^1–5^ Their emissions induce acute/chronic health risks and drive secondary pollution through PM2.5/ozone formation, while paradoxically wasting valuable chemical feedstocks.^6–12^

Available VOC mitigation strategies include destructive methods (*e.g.*, thermal/catalytic combustion) and recovery approaches (*e.g.*, adsorption/absorption).^13–20^ Destructive techniques convert VOCs into CO_*x*_ with carbon penalties, whereas adsorption processes are characterized by flexible regeneration and cost-effectiveness. Therefore, adsorption recovery technology is an economically viable and environmentally friendly approach, with activated carbon adsorption of VOCs being the most extensively studied.^21–26^ However, applications of activated carbon are limited by challenges such as flammability and pore clogging. Thus, bentonite (Bt) has emerged as a superior alternative owing to its (i) layered structure, which enables physisorption/ion-exchange duality; (ii) lower cost than activated carbon, due to the global abundance of Bt raw materials; and (iii) thermal stability during regeneration cycles.^27–29^

Despite these advantages, Bt research has predominantly focused on aqueous-phase applications (*e.g.*, heavy metal removal^30–32^ and wastewater treatment^27,33,34^), whereas its potential for gas-phase VOC adsorption remains underexplored. Conventional porous Bt synthetic routes, such as pillar intercalation and organic template calcination, rely on energy-intensive high-temperature calcination (typically ≥500 °C), which can degrade the framework or generate hazardous emissions.^35,36^ To overcome these limitations, our group developed an ice-templating^37–40^ assembly strategy based on cryogenic structuring principles. This innovative approach uses ice crystals as sacrificial templates that are removed through sublimation during freeze-drying, thereby eliminating the need for high-temperature processing. Although freeze-drying consumes considerable electrical energy for cooling and vacuum generation, this method circumvents the extreme heat demand of calcination. More critically, ice-templating is renowned for constructing well-defined macroporous channels (*e.g.*, tens to hundreds of nanometers).^41^ Coupled with the intrinsic nanoscale pores of exfoliated clay sheets, this process facilitates the formation of a hierarchical architecture. The resulting material enables multiscale VOC capture through synergistic mechanisms: convective gas transport through macropores, Knudsen diffusion within mesopores, and enhanced surface interactions.

We posit that a hierarchical pore architecture in clay-based aerogels can be achieved by combining the intrinsic porosity of the clay layers with the macroporosity created by ice-templating. Specifically, the raw bentonite itself possesses inherent slit-shaped micropores and mesopores arising from its layered crystal structure and interlayer spacing. During the ice-templating process, the controlled exfoliation of these layered units does not eliminate this intrinsic micro/mesoporosity but rather reassembles them into a three-dimensional network. This is distinctly different from the mechanism of calcination, which primarily creates new micropores and mesopores by the thermal decomposition of organic templates.

This study systematically evaluates the adsorption performance of ice-templated Bt using three model VOCs (benzene, toluene, and acetone) that span a range of polarities and molecular sizes. Notably, the design incorporates an abundant primary agricultural product (lotus starch) as a biodegradable crosslinking agent. A comprehensive investigation of key operational parameters (*e.g.*, suspension concentration, VOC feed concentration, flow rate) reveals quantitative structure–activity relationships. This work demonstrates significant industrial potential by providing a sustainable strategy for fabricating clay-based adsorbents from natural agricultural resources. This approach has key advantages over using activated carbon, including its low cost due to the abundance of raw materials, and a synthetic process that avoids the high-temperature calcination required for conventional porous adsorbents. Additionally, this study elucidates previously overlooked molecular-level VOC-clay interactions that are critical for designing high-performance air purification systems.

## 2 Materials and methods

### 2.1 Materials

Raw Bt (Chengdu Shengdi Bentonite Co., Ltd) was subjected to triplicate gravitational sedimentation. Food-grade lotus starch served as the hierarchical porogen. All reagents (Chengdu Kelong Chemical Co.) were of analytical grade and used as received.

### 2.2 Porous material preparation

Purified Bt suspensions (A1–A4) were prepared *via* three-cycle purifications. Briefly, raw Bt (100–250 g) was dispersed in 1000 mL deionized water, homogenized (800 rpm, 40 min), and allowed to settle for 24 h, and colloids were recovered by siphoning. During gel synthesis, ethanol-modified suspensions (10 mL each) were combined with 10% lotus root starch hydrogels (20 mL, gelatinized at 65 °C/800 rpm/2 min). Vortex mixing (30 s) yielded homogeneous composites, which were cast into Φ60 × 40 mm molds. Directional freezing (−15 °C, 2 °C min^−1^) preceded a two-stage lyophilization process; primary (−50 °C, 0.5 °C min^−1^, 20 Pa, 32 h) and secondary drying (20 °C, 16 h) generated porous monoliths (S1–S4). Optimization of the starch concentration indicated that 10% formulations achieved maximal specific surface area with interconnected macropores (>50 µm), whereas 5% and 15% concentrations induced structural collapse and pore occlusion, respectively.

**Fig. 1:**
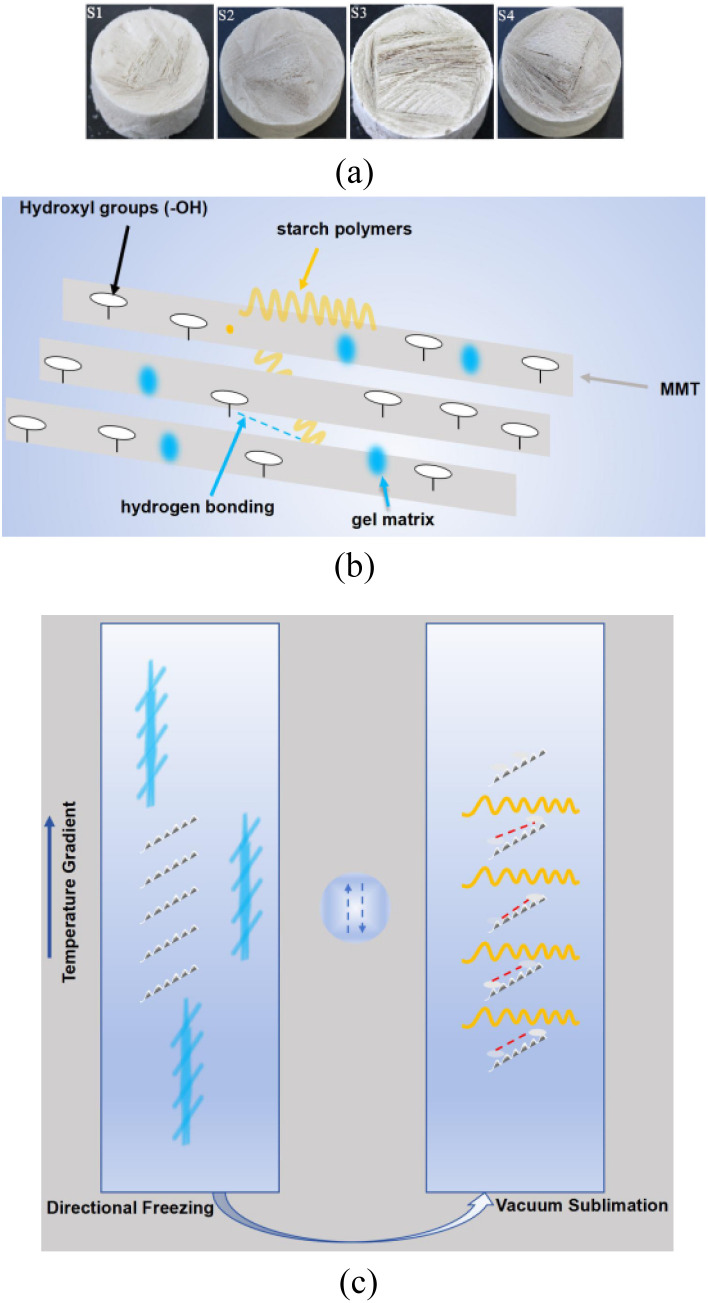
Hierarchical structure and ice-templating process in Bt-montmorillonite (MMT)/starch cryogels: (a) freeze-dried Bt monoliths (S1–S4) across concentrations; (b) MMT/lotus root starch gel network; (c) ice-templating during freeze-drying.

As shown in Fig. 1a, freeze-cast Bt monoliths (S1–S4) demonstrated solid-loading-dependent densification (10–25 wt%) while preserving structural integrity. Lotus root starch acts as a green binder (Fig. 1b), where amylopectin crosslinks exfoliated montmorillonite (MMT) nanosheets (gray) through –OH⋯O hydrogen bonds (blue), forming 3D networks. Unidirectional ice-front growth produced two synergistic effects (Fig. 1c): (i) shear-aligned montmorillonite (001) planes, and (ii) oriented macro- and mesoporous channels formed through ice-matrix confinement.

### 2.3 Material characterization

X-ray diffraction (XRD) analysis was performed using a PW3040/60 diffractometer (Cu-Kα radiation, 20 kV, 50 mA) with a scanning range of 5–80° (5° min^−1^). Microstructural evaluations were conducted using a Hitachi S-4800N SEM at 5.0 kV after gold sputtering (5 nm coating). Textural properties were characterized by N_2_ physisorption at 77 K (using a Rise1030 analyzer). Based on the adsorption data, the specific surface area and pore size distribution were determined using the Density Functional Theory (DFT) model.

#### 2.3.1 Adsorption performance and process configuration

**Fig. 2:**
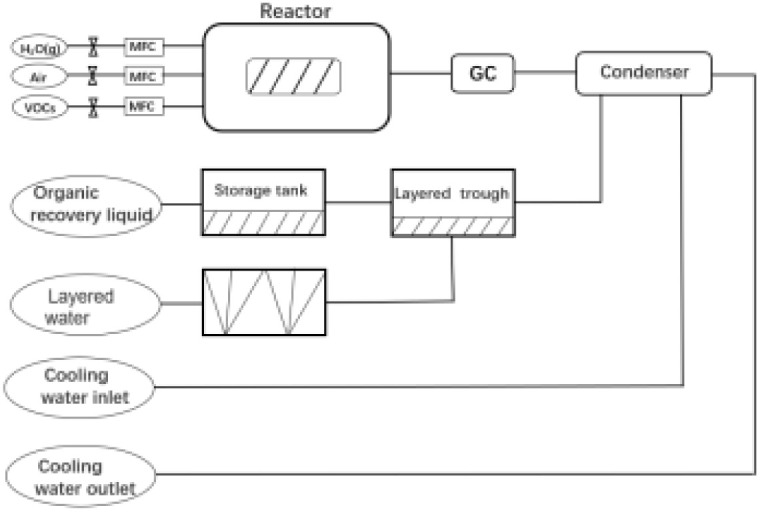
Schematic diagram of bentonite monolith adsorption–desorption cycling.

The gas-phase adsorption system (Fig. 2) incorporated a GC-1690 with FID detector maintained at 300 °C. Cylindrical Bt monoliths (Φ18 × 40 mm) were axially packed in quartz columns (20 × 200 mm) with quartz wool. VOC/N_2_ mixtures (300 ± 10 ppm) were delivered through a mass flow controller at 150 mL min^−1^ under controlled conditions (25.0 ± 0.5 °C, 101.3 ± 0.5 kPa). Adsorption capacities were determined by integrating breakthrough curve areas according to eqn (1),1
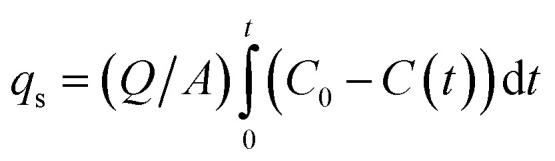
where *Q* is the flow rate (mL min^−1^); *A* is the adsorbent mass (g); and *t* is the breakthrough threshold time (min). The adsorption saturation (*C*/*C*_0_ > 0.95, RSD ≤5% across triplicates) was used to confirm monolayer coverage.

Cyclic stability was assessed through ten consecutive adsorption–desorption cycles using thermal regeneration. The regeneration protocol included a temperature-programmed N_2_ purge (50 mL min^−1^) from 25 to 110 °C at a heating rate of 10 °C min^−1^, sustained until the outlet gas concentration decreased below the detection limit. The capacity retention ratio (*R*_c_) was calculated using eqn (2),

2 *R*_c_(%) = *q*_*n*_/*q*_1_ × 100%

where *q*_*n*_ represents the adsorption capacity at the *n*th cycle.

#### 2.3.2 Methylene blue assay

Methylene blue (MB) adsorption assays were conducted to quantify the MMT content in Bt samples. First, 0.2 g of the sample was dispersed in 50 mL distilled water containing 20 mL of 1% sodium pyrophosphate (Na_4_P_2_O_7_) and boiled for 5 min. After adding a 0.2 wt% MB solution, the mixture was shaken vigorously for 30 s. The adsorption endpoint was determined *via* drop deposition on filter paper until distinct blue halos emerged. The MMT content was calculated using eqn (3) and (4),3
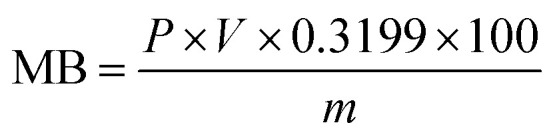
4
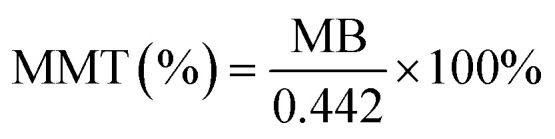
where *P* is the MB solution concentration (g mL^−1^); *V* denotes the titrant volume (mL); *m* represents the sample mass (g); 0.3199 is the molar mass conversion factor for MB; and 0.442 corresponds to the theoretical cation-exchange capacity (CEC) of pure MMT (mmol g^−1^).

## 3 Results and analysis

### 3.1 MB adsorption and XRD characterization

The MB adsorption method can be used to quantify the MMT content in Bt based on its inherent correlations with the CEC and interlayer charge density. Pure MMT samples exhibit batch-dependent adsorption (70–150 mmol/100 g), whereas geological samples demonstrate significant correlations between MB adsorption capacity and MMT content (*R*^2^ > 0.85, *p* < 0.01).^42^ Systematic purification increased the MMT content in raw Bt from 69% to 86.9% (Table 1), thus exceeding the critical threshold of 80% required for functionalization.

**Table 1:** MB absorption test results for Bt(g/100 g)

	Trial 1	Trial 2	Trial 3	Average	MMT (%)
Raw sample	29.8	30.4	31.2	30.5	69
Purified sample	38.3	38.9	37.9	38.4	86.9

**Fig. 3:**
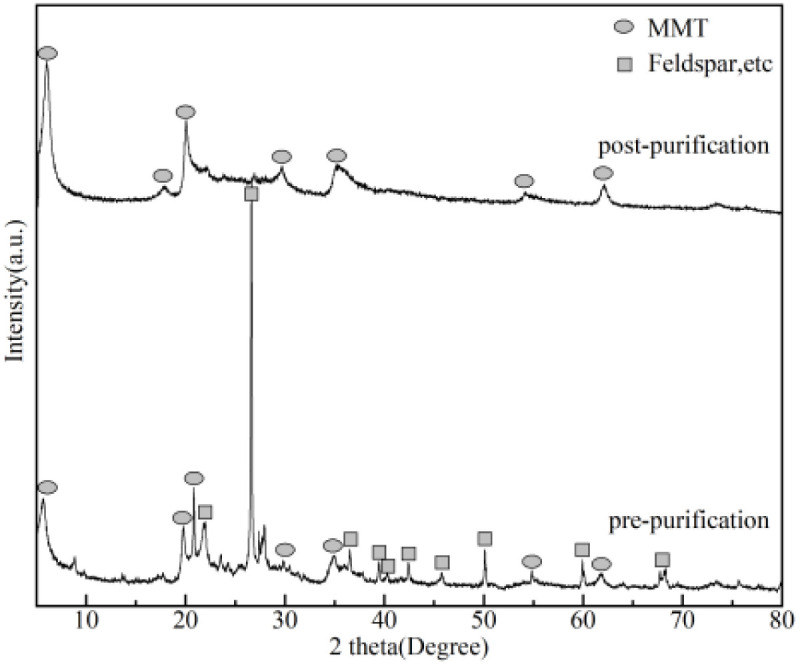
XRD patterns of raw and purified Bt.

XRD analysis provided complementary validation. As shown in Fig. 3, the (001) basal reflection of MMT at 2*θ* = 5.74° intensified significantly, while characteristic peaks, such as that at 2*θ* = 27.32°, nearly disappeared. These observations indicated substantial elimination of impurity phases (*e.g.*, quartz and feldspar) from the raw Bt. This crystallographic purification was consistent with the MB adsorption quantification results, which confirmed an absolute increase of 17.9% in terms of MMT content (Table 1).

### 3.2 SEM and multiscale pore structure evolution

**Fig. 4:**
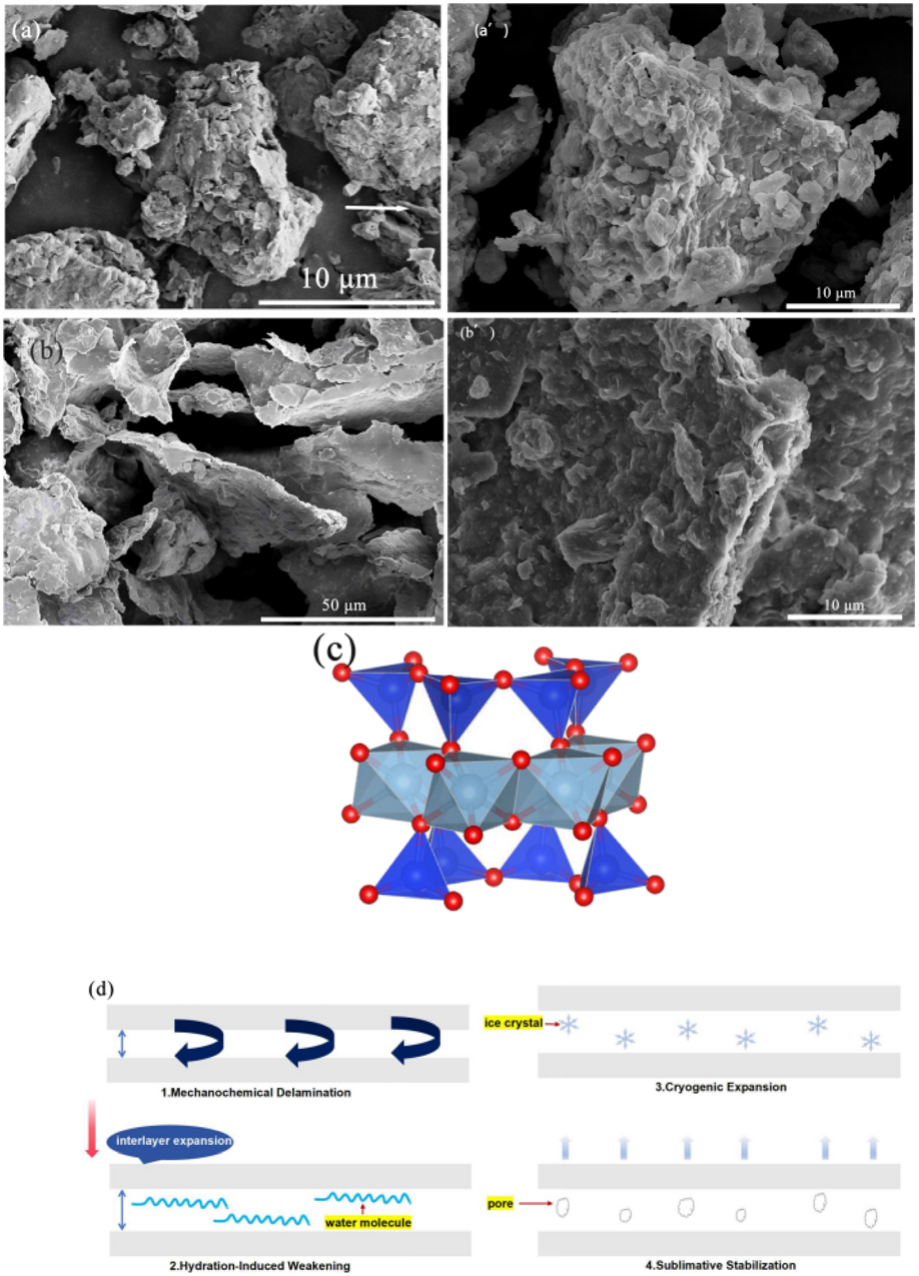
(a and a′) SEM images of raw Bt; (b and b′) SEM images of S3; (c) MMT layered configuration; (d) hierarchical pore formation mechanism.

The intrinsic hierarchical architecture of pristine Bt is illustrated in Fig. 4(a, a′). Low-magnification imaging (Fig. 4a) revealed irregular block-like aggregates containing submicron-scale pores (200–800 nm) and interlayer fractures (arrows). This structure originated from the 2 : 1 layered silicate configuration of MMT (Fig. 4c), where two tetrahedral SiO_4_ sheets coordinate with an octahedral AlO_6_ layer through shared apical oxygen atoms. Interlamellar cohesion is maintained through weak van der Waals forces and charge-balancing cations (Na^+^/Ca^2+^).

Physicochemical modifications induced significant structural reorganization in S3 Fig. 4(b, b′). Compared to pristine Bt, the modified S3 showed disintegration of the original dense aggregates into thinner lamellar structures, with slit-shaped pores clearly visible at fracture surfaces in high-magnification images. These lamellar structures are preferentially oriented along the (001) cleavage plane, constructing a multiscale pore system ranging from microscopic slits to macroscopic channels. Comparative analysis revealed transformed layered sheets that formed an interconnected porous network through bridging configurations with slit-shaped pores, ink-bottle cavities, and spherical mesopores.

The structural evolution mechanism involved four coordinated stages (Fig. 4d). First, high-intensity shear processing generated interfacial shear stress exceeding the interlayer binding energy, which induced basal plane separation and consequential expansion of the interlayer spacing. Subsequent hydration weakened the interlamellar bonding through water intercalation. Controlled freezing (−15 °C) induced ice crystallization, which created anisotropic stress that triggered irreversible delamination. Vacuum lyophilization (−50 °C, 20 Pa) preserved the pore architecture *via* ice sublimation, yielding a superhydrophobic material that could resist capillary collapse. This strategy increased the pore volume by 43.9% (*i.e.*, from 0.082 to 0.118 cm^3^ g^−1^) and enhanced VOC adsorption (refer to Section 3.4).

### 3.3 Specific surface area and pore architecture analysis

Pore structure was characterized using Non-Local Density Functional Theory (NLDFT) with a slit-pore model, which provides superior accuracy for layered clay materials compared to the conventional BET method. NLDFT properly accounts for fluid–solid interactions during nitrogen adsorption at 77 K and simultaneously describes both micropore filling and mesopore capillary condensation in hierarchical pore systems. The NLDFT results are presented in Table 2. The specific surface area increased in the following order: Bt (41.3 m^2^ g^−1^) < S1 (48.7 m^2^ g^−1^) < S2 (58.2 m^2^ g^−1^) < S4 (63.5 m^2^ g^−1^) < S3 (71.3 m^2^ g^−1^), demonstrating the clear efficacy of the modification process.

**Table 2:** Key textural parameters

Sample	DFT surface area (m^2^ g^−1^)	Total pore volume (cm^3^ g^−1^)	Micropore volume (cm^3^ g^−1^)	Mesopore volume (cm^3^ g^−1^)	Macropore volume (cm^3^ g^−1^)
Bt	41.3	0.082	0.001	0.078	0.003
S1	48.7	0.089	0.001	0.083	0.005
S2	58.2	0.106	0.001	0.097	0.008
S3	71.3	0.118	0.002	0.095	0.021
S4	63.5	0.112	0.001	0.098	0.013

This optimized mesostructure enhanced the surface area of S3 by 1.7-fold compared to pristine Bt, while also increasing the total pore volume to 0.118 cm^3^ g^−1^. Crucially, NLDFT analysis confirmed only minimal development of microporosity (0.001–0.002 cm^3^ g^−1^), indicating that the increase in surface area originated mainly from mesostructural optimization rather than the formation of new micropores. The limited micropores detected are primarily inherent to the raw bentonite material.

**Fig. 5:**
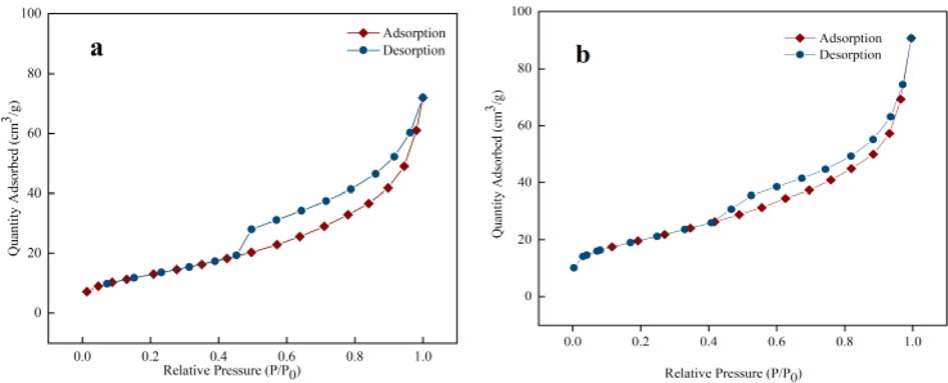
(a and b) N_2_ adsorption–desorption isotherms for raw bentonite and S3, respectively.

Isotherm analysis (Fig. 5) further supported these findings. The pristine Bt sample exhibited a Type IV isotherm with a broad H3-type hysteresis loop (Fig. 5a). In contrast, S3 showed an extended hysteresis loop across the *P*/*P*_0_ range of 0.4–1.0, and its adsorption capacity at high relative pressure (*P*/*P*_0_ > 0.9) increased by approximately 40% compared to Bt (Fig. 5b). This enhancement is attributed to the development of an ice-templated macropore network, as evidenced by the increase in macropore volume from 0.003 cm^3^ g^−1^ (Bt) to 0.021 cm^3^ g^−1^ (S3). Sample S4 exhibited an intermediate macropore volume of 0.013 cm^3^ g^−1^, suggesting only partial development of the macroporous network.

When the optimal modification conditions were exceeded (as in S4), the surface area stabilized at 63.5 m^2^ g^−1^–11% lower than that of S3-indicating a critical threshold in precursor concentration (approximately 16.7 wt% Bt). Excessive layered units introduced structural reorganization defects, which compromised pore uniformity.

The structural evolution follows a three-stage mechanism: interlayer exfoliation removes lattice distortions; ice templating constructs a macroporous network that reduces diffusion resistance; and optimized sheet assembly concentrates porosity within efficient adsorption ranges. The observed performance hierarchy (S3 > S4 > S2 > S1 > Bt) confirms a progressive optimization process, with S3 representing the ideal balance among exfoliation, templating, and reassembly.

### 3.4 Adsorption performance

#### 3.4.1 Toluene adsorption

**Fig. 6:**
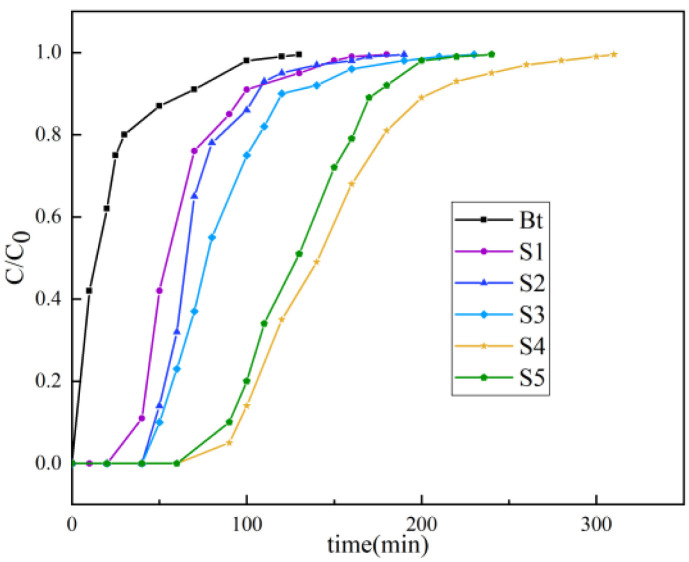
Toluene breakthrough curves of different samples.

The toluene adsorption characteristics were systematically evaluated based on breakthrough curve measurements under controlled conditions (298 K, 1 atm, 150 mL min^−1^ flow rate, 300 ppm inlet concentration). The two critical parameters were the breakthrough time (*t*_b_; outlet concentration reaching 5% of the inlet concentration) and saturation time (*t*_s_; outlet/inlet concentration ratio approaching 1). As shown in Fig. 6, modified adsorbents exhibited enhanced performance relative to pristine Bt, with equilibrium capacities descending as follows: S3 (53.8 mg g^−1^) > S4 (46.2 mg g^−1^) > S2 (36.2 mg g^−1^) > S1 (31.8 mg g^−1^) >> Bt (9.4 mg g^−1^).

Pristine Bt demonstrated immediate toluene breakthrough (*t*_b_ < 1 min) due to ineffective physisorption, whereas the modified samples showed progressive breakthrough delay, with S3 achieving the longest *t*_b_ of 90 min. The strong linear correlation between the adsorption capacity and BET surface area confirmed that surface accessibility is the dominant adsorption driver.

The superior performance of S3 originated from molecular dimension matching (toluene kinetic diameter = 0.67 nm), enhanced van der Waals interactions in nanoscale channels, and transport through the hierarchical porous structure. The reduced capacity of S4 was likely due to precursor concentration effects during slurry preparation, where excessive Bt loading may have induced incomplete structural reorganization, thereby compromising the framework integrity and active interface availability.

#### 3.4.2 Concentration-dependent adsorption kinetics

**Fig. 7:**
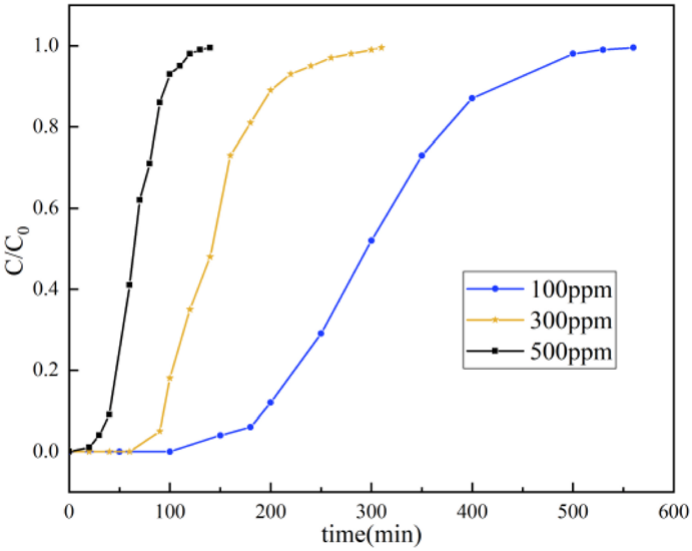
Breakthrough curves of toluene on S3 adsorbent at various inlet concentrations.

The effects of the toluene inlet concentration (100–500 ppm) on the adsorption performance of S3 are presented in Fig. 7. The concentration effect was governed by two competing mechanisms. On one hand, a higher concentration increases the collision frequency of toluene molecules with active sites (following gas molecular kinetics). This increases the equilibrium adsorption capacity from 51.2 mg g^−1^ (100 ppm) to 54.6 mg g^−1^ (500 ppm), representing an enhancement of approximately 6.6%. On the other hand, at higher concentrations, high-affinity sites are rapidly occupied, while the boundary layer effect intensifies. This can lead to reduced pore accessibility, which manifests as a significantly shorter breakthrough time. The adsorption process fit a pseudo-second-order kinetic model. Notably, as the concentration increased from 100 to 500 ppm, the adsorption rate constant increased correspondingly, thus further confirming that mass transfer resistance (particularly external diffusion) is the main factor limiting the adsorption process.

The hierarchical pore structure of S3 effectively mitigated the constraints of high concentrations through multi-scale synergistic effects. Macropores (>50 nm) acted as efficient gas-phase transport channels, which accelerated molecular diffusion into the material interior. S3 provided high specific surface area to maintain a high density of adsorption sites. Simultaneously, surface hydrophobicity effectively suppressed competitive adsorption by water molecules, thereby ensuring selective capture of toluene. These synergistic multi-level pore structures and surface properties endowed S3 with the unique ability to balance adsorption capacity and adsorption kinetics (*i.e.*, adsorption rate and breakthrough time) over a wide concentration range (100–500 ppm). These results highlight a novel material design strategy for the efficient capture of VOCs.

#### 3.4.3 Molecular polarity-dependent adsorption selectivity mechanism

**Fig. 8:**
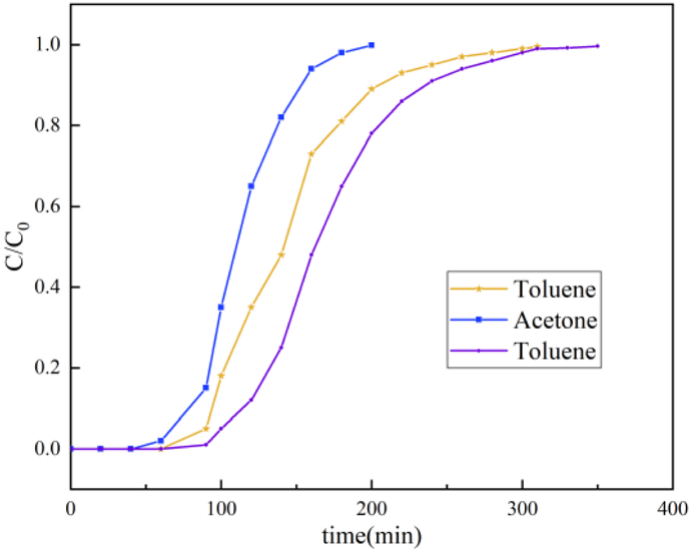
Breakthrough curve for the adsorption of various VOCs.

As shown in Fig. 8, under standardized conditions (300 ppm, 150 mL min^−1^ flow rate, 298 K), the optimized material S3 exhibited distinct equilibrium adsorption capacities for the following representative VOCs: acetone (54.3 mg g^−1^) > toluene (53.8 mg g^−1^) > benzene (46.2 mg g^−1^). This polarity-driven selectivity arose from interactions between the molecular properties of VOCs and the structural features of MMT. The polar surface groups (–OH, Si–O^−^) and exchangeable Na^+^ cations in MMT preferentially adsorbed highly polar acetone through ion–dipole interactions, whereas nonpolar benzene relied solely on weaker van der Waals forces. Toluene's intermediate adsorption strength was attributed to methyl-induced π-electron cloud distortion, which generated mild polarity.

These mechanistic insights support a two-pronged engineering strategy to enhance practical performance. First, surface cation engineering (*e.g.*, Ca^2+^ doping) can amplify polar-site density, which could enable targeted enhancement of the material's acetone capture efficiency. Second, the material's superhydrophobic surface can effectively mitigate competitive water adsorption in humid environments, thus addressing the humidity sensitivity of conventional adsorbents. This innovative integration of a polarity-gradient design and hydrophobic shielding can be applied to optimize VOC separation specificity and to ensure operational stability under real-world conditions. These findings establish a new paradigm for advanced adsorbent development.

#### 3.4.4 Thermal regeneration and adsorption performance

**Fig. 9:**
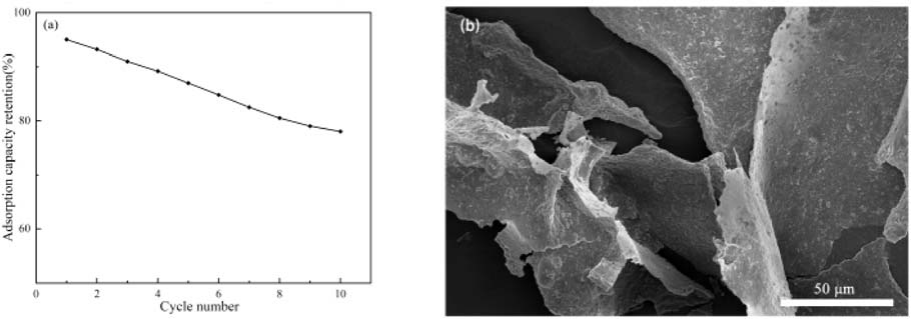
(a) Reusability of S3 (ten cycles); (b) SEM images of the S3 after the VOC adsorption test.

The cycling stability of the optimal S3 porous material was assessed through ten successive adsorption–desorption runs (Fig. 9a). A retention of 78% of the initial adsorption capacity was observed, indicating satisfactory recyclability. To provide a meaningful performance benchmark, comparisons were drawn with advanced adsorbents documented elsewhere. For example, a structured rGO/MMT/XDV aerogel containing synthetic hyper-crosslinked polymers reported a regeneration ratio over 96% across 5 cycles.^43^ In another case, commercial activated carbon subjected to vacuum swing adsorption-under thermal (363.15 K) and vacuum (13 332 Pa) conditioning-preserved approximately 90% of its uptake after 5 cycles.^44^

Although the capacity retention of S3 is numerically lower, these comparisons reveal a deliberate trade-off in adsorbent design philosophy. The higher regeneration ratios of the reference materials are attained through the use of expensive functional constituents or energy-demanding regeneration setups. By contrast, the present S3 material is derived entirely from natural and low-cost precursors (lotus starch and bentonite) *via* a facile ice-templating process. Its 78% capacity retention after 10 cycles is achieved under mild thermal regeneration, requiring no specialized equipment or considerable energy expenditure. Post-cycling SEM characterization (Fig. 9b) further verified the retention of the material's porous architecture, confirming mechanical and structural robustness over repeated use.

In light of these attributes, the S3 adsorbent demonstrates a compelling combination of sustainable precursor sourcing, mild-regeneration capability, and consistent cyclability. It thus represents a practical and resource-efficient option for VOC treatment scenarios where operational simplicity and low life-cycle cost are prioritized.

#### 3.4.5 Hierarchical adsorption mechanism

**Fig. 10:**
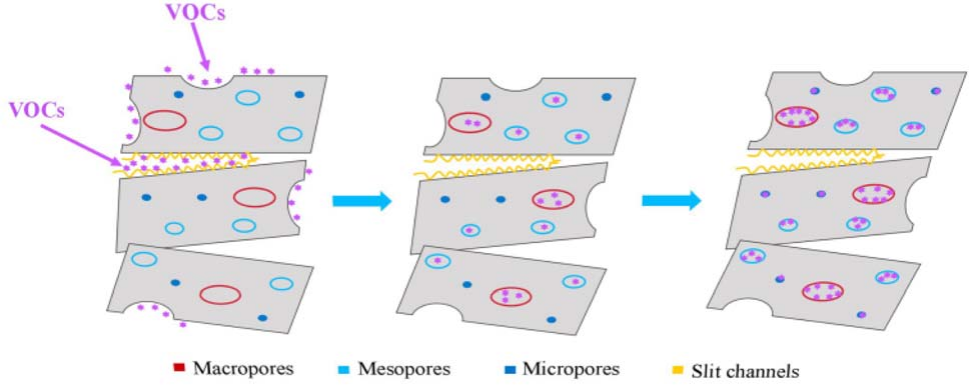
Multiscale adsorption mechanism in ice-templated bentonite.

Fig. 10 shows the multiscale adsorption process in ice-templated Bt composites featuring a hierarchical porous network dominated by mesopores (2–50 nm) with coexisting macropores (>50 nm), micropores (<2 nm), and interlamellar slits.

VOC adsorption on these composites occurred through three synergistic phases. Initial physisorption on external surfaces and slit pores (driven by van der Waals forces and surface accessibility) transitioned to pore-specific transport mechanisms. Macropores facilitated gas-phase molecular diffusion, while mesopores enabled Knudsen diffusion governed by molecule-wall collisions. Micropores (<2 nm) induced configurational diffusion through continuous molecular confinement, which enabled multilayer adsorption *via* combined physisorption and cation-mediated chemisorption (*e.g.*, Na^+^ coordination, hydroxyl bonding).

Experimental data revealed three key factors in addition to surface area. First, molecular polarity enhanced adsorption through ion–dipole interactions with interlayer cations, while also reducing water competition. Second, the interconnected hierarchical pores balanced molecular transport and adsorption-site density. Third, pore confinement and cation selectivity dictated chemisorption efficiency. The ice-templated architecture uniquely promotes a high surface area, optimized pore hierarchy, and numerous active sites, thus affording higher polar VOC capacities than conventional adsorbents. This multiscale synergy involving molecular interactions and pore engineering explains the material's exceptional performance for practical VOC capture.

#### 3.4.6 Comparative performance analysis and material positioning

To place the performance of our lotus starch–bentonite porous material in a broader context, a comparative analysis with representative adsorbents reported in the literature is instructive (see Table 3). As summarized, advanced materials such as activated carbon fibers (ACFs), organic polymers, and activated coconut shell exhibit superior VOC adsorption capacities-often exceeding 500 mg g^−1^ under specific conditions-along with high specific surface areas ranging from 1020 to 3300 m^2^ g^−1^, as reported in previous studies. Similarly, the cyclic stability of our material (78% retention after 10 cycles) is moderate compared with some specially designed composites regenerated under optimized protocols.

**Table 3:** Summary of physiochemical properties and performance of different porous materials for VOC adsorption

Adsorbent	Specific surface areas (m^2^ g^−1^)	Total pore volume (cm^3^ g^−1^)	Adsorption capacity (mg g^−1^)	References
ACs	830–960	0.4–0.5	51–150	45–48
Biochars	1100–3800	0.48–2.5	25–300	49–52
ACFs	1662	0.108	105–540	53 and 54
Graphenes	292.6		344.4	55
Zeolites	334–1081	0.13–1.0	1.72–184	56 and 57
MOFs	1400–2100	1.22–1.23	224–228	58 and 59
Clays	69–330	0.119–0.554	90–142	60 and 61
Silica gel	765.6	0.444	437	62
Organic polymers	1020–3300	1.75–1.78	389–1395	63–65
Composites	348–1112	0.365	116–246	66 and 67

However, this comparison reveals a fundamental divergence in material design strategy. The outstanding performance of the referenced benchmark materials is typically achieved through energy-intensive processes, such as high-temperature calcination, acid activation, or the use of expensive synthetic precursors. In contrast, the distinguishing strength of our material lies in its exceptional sustainability and economic feasibility. It is fabricated from abundant, natural, and non-toxic raw materials-lotus starch and bentonite-via a simple ice-templating process that entirely avoids harsh chemical treatments or high-temperature stages. Although the absolute adsorption capacity of our material is moderate, it remains competitive among clay-based and many biomass-derived adsorbents, even outperforming several in its category.

Thus, this work proposes a new paradigm that emphasizes a balanced compromise among performance, cost, and environmental impact. Our porous material is not designed to outperform all high-performance synthetic adsorbents in every metric. Rather, it offers a practical and eco-friendly alternative for large-scale, cost-driven environmental applications where ease of operation, low regeneration energy input, and a minimal ecological footprint are critical considerations.

The abbreviations in the table represent the following types of adsorbents: ACs (Activated Carbons), Biochars (Biochar), ACFs (Activated Carbon Fibers), and MOFs (Metal–Organic Frameworks). The data in Table 3 also include other materials such as graphene, zeolites, and clays for comparison. It is noteworthy that activated coconut shell-based material serves as a special case among biochars, with reported adsorption capacities significantly exceeding 1700 mg g^−1^ ref. 68 and 69-far above the conventional biochar range-highlighting the critical role of specific precursors and activation techniques in determining performance.

## 4 Conclusions

This study establishes an ice-templated self-assembly strategy for fabricating hierarchical porous bentonite adsorbents, which exhibit structural and functional enhancements over raw Bt and commercial activated carbon materials. The optimized architecture features lamellar-bridging networks with slit-shaped mesopores, a 71.3 m^2^ g^−1^ specific surface area, and a 0.118 cm^3^ g^−1^ pore volume. These structural advancements provide exceptional VOC capture performance, with toluene capacity reaching 53.8 mg g^−1^ (4.7× improvement) and a polarity-driven selectivity hierarchy: acetone > toluene > benzene. The adsorption mechanism involves a cascading process including macropore convection, mesopore Knudsen diffusion, and micropore configurational diffusion, supported by physisorption (van der Waals) and chemisorption (cation complexation) interactions.

The developed material demonstrates significant industrial application potential. Its main advantages over conventional activated carbon adsorbents include (i) low raw material costs due to the wide availability of Bt, (ii) reduced energy consumption during synthesis by applying non-calcination processing, and (iii) strong operational cost-efficiency due to inherent material characteristics enabling low-energy regeneration and humidity resistance. Future research efforts should focus on three priorities: precise pore engineering by regulating ice-crystal dynamics, enhancing affinity for nonpolar VOCs *via* metal doping, and pilot-scale validation. The sustainable clay-based adsorbent paradigm established herein provides a solution for developing next-generation low-energy air pollution control systems.

While this study demonstrates excellent VOC adsorption at the tested concentrations, the performance at trace-level (*e.g.*, sub-ppm) concentrations is a critical metric for practical air purification. The highly accessible mesoporous structure and large specific surface area of our porous material are anticipated to facilitate effective diffusion and capture of low-concentration VOCs. Future research efforts will therefore focus on: (1) evaluating adsorption isotherms at parts-per-billion levels and determining the minimum detectable levels for a range of VOCs; (2) precise pore engineering by regulating ice-crystal dynamics; and (3) pilot-scale validation. The sustainable clay-based adsorbent paradigm established herein provides a foundational solution for developing next-generation, low-energy air pollution control systems.

## Conflicts of interest

There are no conflicts to declare.

## Data Availability

The data that support the findings of this study are available within the article and are not additionally included in any Supplementary information files.
